# Sex Differences in the Feasibility of Aerobic Exercise Training for Improving Cardiometabolic Health Outcomes in Adults with Type 2 Diabetes

**DOI:** 10.3390/jcm12041255

**Published:** 2023-02-04

**Authors:** Sian Alice O’Gorman, Clint Thomas Miller, Jonathan Charles Rawstorn, Angelo Sabag, Rachelle Noelle Sultana, Sean Michael Lanting, Shelley Elizabeth Keating, Nathan Anthony Johnson, Kimberley Larisa Way

**Affiliations:** 1 Faculty of Health, School of Exercise and Nutrition Sciences, Deakin University, Burwood, VIC 3125, Australia; 2 Institute for Physical Activity and Nutrition, School of Exercise and Nutrition and Sciences, Deakin University, Geelong, VIC 3220, Australia; 3 NICM Health Research Institute, Western Sydney University, Campbelltown, NSW 2560, Australia; 4 Faculty of Medicine and Health, Discipline of Exercise and Sport Science, University of Sydney, Sydney, NSW 2006, Australia; 5 Faculty of Health and Medicine, School of Health Sciences, University of Newcastle, Newcastle, NSW 2300, Australia; 6 School of Health Sciences, Western Sydney University, Campbelltown, NSW 2560, Australia; 7 School of Human Movement and Nutrition Sciences, The University of Queensland, Brisbane, QLD 4072, Australia; 8 Charles Perkins Centre, University of Sydney, Camperdown, NSW 2560, Australia; 9 Exercise Physiology and Cardiovascular Health Lab., Division of Cardiac Prevention and Rehabilitation, University of Ottawa Heart Institute, Ottawa, ON K1Y 4W7, Canada

**Keywords:** feasibility, sex differences, arterial health, pulse wave velocity, aerobic exercise training

## Abstract

Females with type 2 diabetes (T2D) have a 25–50% greater risk of developing cardiovascular disease compared with males. While aerobic exercise training is effective for improving cardiometabolic health outcomes, there is limited sex-segregated evidence on the feasibility of aerobic training in adults with T2D. A secondary analysis of a 12-week randomized controlled trial examining aerobic training in inactive adults with T2D was conducted. Feasibility outcomes were recruitment, retention, treatment fidelity, and safety. Sex differences and intervention effects were assessed using two-way analyses of variances. Thirty-five participants (14 females) were recruited. The recruitment rate was significantly lower among females (9% versus 18%; *p* = 0.022). Females in the intervention were less adherent (50% versus 93%; *p* = 0.016), and experienced minor adverse events more frequently (0.08% versus 0.03%; *p* = 0.003). Aerobically trained females experienced clinically meaningful reductions in pulse wave velocity (−1.25 m/s, 95%CI [−2.54, 0.04]; *p* = 0.648), and significantly greater reductions in brachial systolic pressure (−9 mmHg, 95%CI (3, 15); *p =* 0.011) and waist circumference (−3.8 cm, 95%CI (1.6, 6.1); *p* < 0.001) than males. To enhance the feasibility of future trials, targeted strategies to improve female recruitment and adherence are needed. Females with T2D may experience greater cardiometabolic health improvements from aerobic training than males.

## 1. Introduction

Type 2 diabetes (T2D) is a progressive disease characterized by insulin resistance, impaired insulin secretion (ß-cell dysfunction), and subsequent hyperglycemia [1]. Global prevalence has more than doubled over the past three decades and is projected to exceed 570 million by 2025 [2]. Direct (i.e., healthcare) and indirect costs (e.g., absenteeism, reduced productivity, early retirement) were US $1.32 trillion in 2015, and are forecast to reach US $2.48 trillion by 2030 [3]. For people with T2D, cardiovascular disease (CVD; e.g., stroke, myocardial infarction) is the leading cause of mortality and a major contributor to the T2D burden [4,5]. While non-diabetic pre-menopausal females have a lower CVD risk than male counterparts, this advantage is lost in pre-menopausal females with T2D. Females with T2D have a 25–50% greater risk of developing CVD than males [6,7,8], and have poorer outcomes, including higher healthcare expenditures [9], and increased mortality [8], independent of age. This suggests existing T2D management strategies do not adequately address the increased CVD risk and burden among females with T2D. While aerobic exercise training is a first-line management strategy for T2D and CVD [1], females have historically been under-represented in clinical trials [10]. Therefore, it is difficult to determine the uptake of such interventions across sexes. As females with T2D are ~65% less likely than male counterparts to meet physical activity guidelines [11], it is important to examine the feasibility of aerobic training interventions to identify potential sex-specific barriers and considerations that may influence participant recruitment and engagement.

There are sex differences in responses to aerobic training among healthy individuals, e.g., females demonstrate greater vasodilation and reliance on increased peripheral oxygen extraction to compensate for smaller increases in cardiac output [12,13]. However, it is unclear if there are sex differences in training responses in adults with T2D. Sex differences are evident in pathological T2D-induced vascular changes [14], which may influence training responses. Understanding sex-specific responses to training could enhance the tailoring of exercise prescription to optimise health outcomes for both sexes and offset the high burden of disease experienced by females. However, to date, the feasibility of investigating sex-specific aerobic exercise training responses has not been examined in people with T2D. Therefore, the primary aim of this secondary analysis was to identify sex-specific barriers and risks that may influence recruitment, retention, treatment fidelity, and safety of aerobic training interventions in adults with T2D. As data on potential sex differences in exercise training responses is limited, an additional exploratory aim was to describe preliminary sex differences in response to aerobic training on arterial stiffness and other cardiometabolic health outcomes in this population.

## 2. Materials and Methods

### 2.1. Study Design

This was a secondary analysis of data from a 12-week three-arm parallel randomized controlled trial comparing effects of aerobic exercise training versus an exercise placebo control on cardiometabolic risk factors in adults with T2D and obesity [15,16]. Participants were randomly allocated to complete three weekly sessions of: (i) low-volume high-intensity training; (ii) moderate-intensity continuous training; or (iii) placebo exercise control. A computer-generated simple random allocation sequence was concealed by opaque sequentially numbered envelopes and administered by a blinded study investigator following baseline data collection. All training sessions and assessments were undertaken at the Charles Perkins Centre, University of Sydney. The study protocol conformed to the ethical guidelines of the 1975 Declaration of Helsinki and was approved by the Human Research Ethics Committee of the University of Sydney (2014/961) and Royal Prince Alfred Research Ethics and Governance Office. The trial was prospectively registered with the Australian New Zealand Clinical Trial Registry (ANZCTR: 12614001229651).

### 2.2. Recruitment and Eligibility

Participants were recruited via a database and advertisements displayed at the Charles Perkins Centre, Royal Prince Alfred Hospital, and the University of Sydney website. Eligible participants were adults (18–65 years) with medically diagnosed T2D, a BMI of 30–45 kg/m^2^, and were physically inactive (self-reported <150 min/week or <three days/week). Exclusion criteria included: uncontrolled diabetes, unstable cardiac condition, and inability to commit to a 12-week training program. Participants provided written informed consent before being medically cleared for participation.

### 2.3. Interventions

All exercise sessions were supervised by experienced exercise professionals. Exercise was performed on electronically-braked upright cycle ergometers (Corival, Lode, The Netherlands). Heart rate, blood pressure, and Borg’s rating of perceived exertion (RPE) [17] were monitored throughout. Blood glucose was measured before and after exercise, and during exercise if signs or symptoms of hypoglycemia presented. If blood glucose was ≤4 mmol/L, 20 g of fast-acting carbohydrates were administered and blood glucose was remeasured after 10–15 min, and was repeated until blood glucose returned above 4 mmol/L.

#### 2.3.1. Aerobic-Exercise

Moderate-intensity continuous training progressively increased from 30 to 45 min over the first 4 weeks, at a workload equivalent to 60%VO_2peak_. Sessions included a 5-min warm-up and cooldown at 50%VO_2peak_ for a total of 55 min per session by Week 4. Low-volume high-intensity training comprised one 4-min bout at a workload equivalent to 90%VO_2peak_, which included a 10-min warm-up and 5-min cooldown at 50%VO_2peak_ for a total of 19 min per session. If unable to complete the 4-min bout, participants were encouraged to cycle until volitional fatigue. By Week 4, all participants were able to complete 4 min. As both interventions were found to be similarly effective in the primary trial analysis [15,16], data were pooled for this secondary analysis to enhance statistical power.

#### 2.3.2. Placebo Exercise Control

Participants were prescribed static leg, chest, arm, and back stretches, and floor-based Pilates exercises (transverse abdominis, internal/external obliques, gluteal muscles). Participants performed 5 min cycling at low intensity (20 W) before and after each session to maintain familiarity with the ergometer. Sessions lasted 30 min and were undertaken fortnightly.

### 2.4. Data Collection

Data were collected at baseline and 12 weeks (≥72 h and ≤7 days after the final session). Participants were asked to arrive in a fasted state (≥10 h), abstain from alcohol and strenuous exercise for 24 h, and refrain from smoking and taking usual prescription medicine the morning of data collection visits. Participants were requested to record their evening meal prior to baseline assessment for replication at follow-up.

### 2.5. Primary Outcome

#### Feasibility

Outcomes to determine the feasibility of the aerobic training intervention based on sex are described in Table 1. Feasibility was defined a priori as no serious adverse events plus two of the following: (i) ≥50% eligible participants recruited; (ii) ≥70% recruited participants retained; and (iii) ≥80% global adherence. Adverse events were defined as any untoward incident during or immediately pre- and post-exercise [18], and considered serious if they were: (i) life-threatening or resulted in death; (ii) required medical treatment; or (iii) resulted in significant disability or incapacity.

### 2.6. Exploratory Outcomes

#### 2.6.1. Pulse Wave Analysis and Central Hemodynamic Responses

Participants lay supine in a quiet, thermoneutral room (22–24 °C) with the pressure cuff on the right arm. After 5 min, central pressures (systolic [CSP], diastolic [CDP], pulse pressure [PP]), augmentation index (AIx), AIx normalized to a heart rate of 75 bpm (AIx@75bpm), heart rate, and brachial pressures (systolic [SBP], diastolic [DBP]) were measured (SphygmoCor XCEL Version 1.3; AtCor Medical, Sydney, Australia). Measures were repeated if they did not pass the SphygmoCor XCEL quality check.

#### 2.6.2. Pulse Wave Velocity

Immediately following pulse wave analysis, carotid-femoral pulse wave velocity (PWV) was recorded. A pressure cuff was placed as superior as possible on the right upper thigh and a tonometer placed on the right carotid pulse. PWV distance was calculated from measurements of: (i) right carotid pulse to sternal notch; (ii) sternal notch to the top of femoral pressure cuff; and (iii) right inguinal fold to the top of femoral pressure cuff [16]. Measures were repeated if they did not pass the SphygmoCor XCEL quality check. If a recording was unsuccessful on the right side, the left side was attempted.

#### 2.6.3. Anthropometry

Height was measured via stadiometer. Waist circumference was measured to the nearest 0.5 cm at the umbilicus after deep expiration. Body mass was measured using a digital scale (Tanita BC-418; Tanita Corporation, Tokyo, Japan). Body mass index (BMI) was calculated as body mass (kg) divided by height squared (m^2^). Mean outcomes were calculated from triplicate measurements.

#### 2.6.4. Cardiorespiratory Fitness

Participants undertook a graded maximal exercise test on an electronically-braked cycle ergometer. After a 3-min warm-up (females: 35 W, males: 65 W), power output increased by 25 W at each stage (150 s) until volitional fatigue. Heart rate, blood pressure, and RPE were recorded at the end of each stage. VO_2peak_ was calculated from the highest 10 s average of breath-by-breath respiratory gas-analysis data from the final stage (Ultima PFX, MGC Diagnostics, Saint Paul, MN, USA). Maximal effort was determined by a respiratory exchange ratio ≥1.1, RPE ≥17, and/or plateau in VO_2_. Termination criteria included: unable to maintain pedaling within 5 rpm of average cadence, participant request, or adverse cardiovascular responses as per American Heart Association guidelines [19].

#### 2.6.5. Blood Sampling and Analysis

Venous blood was collected from the antecubital vein by a blinded researcher and stored at 4 °C for 2–3 h before analysis by an accredited lab (Douglas Hanley Moir Pty Ltd., Sydney, Australia) for fasting glucose and insulin concentrations, glycated hemoglobin (HbA1c), lipids (triglycerides, total cholesterol, high- and low-density lipoprotein cholesterol [HDL, LDL], and high-sensitivity C-reactive protein (hsCRP).

#### 2.6.6. Resting Heart Rate and Blood Pressure

After 10–15-min of quiet sitting, blood pressure was recorded as the highest of two measures on each arm prior to any other measures. A third measure was taken if initial measures differed by ≥10 mmHg. Heart rate was recorded as the mean of triplicate measures via palpation of the radial pulse.

### 2.7. Statistical Analysis

All data were analyzed with Stata BE/17 statistical software (StataCorp, College Station, TX, USA) using an intention-to-treat approach. Missing or incorrect data were imputed using sex-stratified group mean change scores [20]. Normality and equality of variances were assessed using the Shapiro–Wilk and Levene’s test, respectively. Feasibility outcomes were calculated as per Table 1. If multiple reasons for non-participation were reported, each were included in the analysis to provide a comprehensive understanding of recruitment challenges. Declined participation included individuals who could not be contacted further. Sex differences in frequencies were assessed by two proportion z-tests.

Descriptive statistics for baseline and 12-week change in outcome measures were stratified by sex and treatment group. Continuous data are presented as mean ± standard deviation (descriptive statistics) or 95% confidence intervals (inferential statistics) to provide information about the direction and magnitude of effect [21]. Categorical data are presented as frequencies and proportions (%). Independent samples t-test and Pearson’s chi-squared test were used to assess sex differences in continuous and categorical baseline characteristics, respectively. Sex differences and intervention effects were assessed by two-way analyses of variances (ANOVA), with bootstrapping (1000 resamples) on variables that violated assumptions of normality or homoscedasticity (*n* = 11) [22]. Statistical significance was accepted as *p* < 0.05.

## 3. Results

### 3.1. Baseline Characteristics

**Figure 1:**
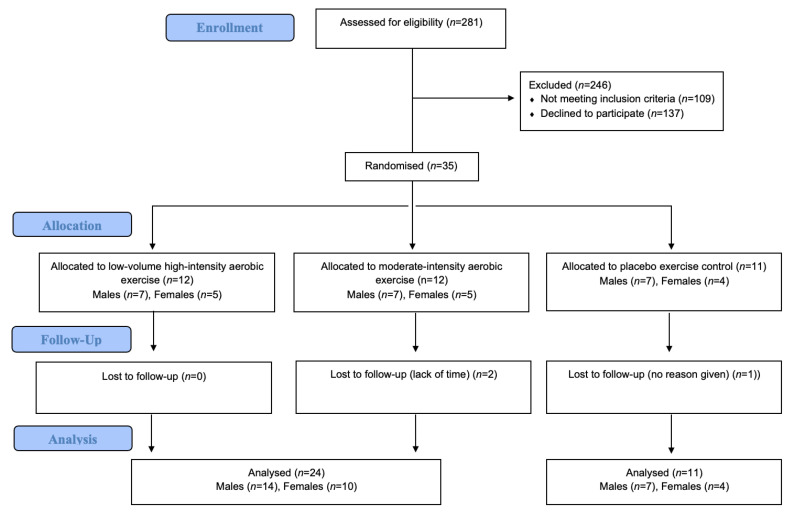
CONSORT flow diagram.

Thirty-five eligible and consenting participants (14 females) were randomized (Figure 1). The cohort had a collective mean age and BMI of 55 ± 1 years and 36.1 ± 0.8 kg/m^2^, respectively (Table 2). Medication data was missing for two participants. Biguanides were the most common anti-hyperglycemic medication (*n* = 30), followed by sulphonylureas (*n* = 8), insulin (*n* = 5), and SGLT2 and DPP-4 inhibitors (both *n* = 3). Eleven participants were taking two or more anti-hyperglycemics to control their T2D. A higher proportion of males were taking anti-hypertensives (71% versus 36%; *p* = 0.036). During the study, one male in the placebo exercise control group began lipid-lowering medication; no other medication changes were recorded. Body mass and VO_2peak_ were 17.7 kg (95%CI [7.3, 28.1]; *p* = 0.002) and 5.2 mL/kg/min (95%CI [2.5, 8.0]; (*p* < 0.001) higher among males, respectively. Baseline AIx (Table 3) and hsCRP (Table 4) were −8.7% (95%CI [−16.6, −0.7]; *p* = 0.033) and −5.11 mg/L (95%CI [−8.41, −1.80]; *p* = 0.004) lower among males, respectively.

### 3.2. Feasibility Outcomes

#### 3.2.1. Recruitment and Retention

A greater proportion of screened females were ineligible or declined participation when compared to males (*p* = 0.022) (Table 5). Subsequently, only 9% were randomized compared to 18% of males. Two or more reasons for non-participation were reported on 18 occasions. For both sexes, the most frequent reasons for non-participation were inability to commit to three sessions per week (male: 13%, female: 20%), disinterest (male: 13%, female: 14%), and living too far from the training facility (male: 10%, female: 12%). No significant sex differences were observed for reasons for non-participation. Retention of randomized participants, from baseline to 12-week follow-up, was 91%. Two participants (one male, one female) in the intervention were lost to follow-up due to lack of time. One female participant in the control group provided no reason for discontinuing the study.

#### 3.2.2. Treatment Fidelity

Males and females in the control group attended 58% (range: 17–100%) and 50% (range: 17–83%) of sessions, respectively. Males and females in the intervention attended 90% (range: 14–100%) and 81% (range: 31–100%) of sessions. Mean compliance was significantly lower among females than males (females: 80%, range 0–100%; males: 99%, range 92–100%; *p* = 0.004), although this may be largely explained by very low compliance among two females (0% and 12%). Consequently, global adherence to the exercise intervention was markedly higher among males (93% versus 50%, *p* = 0.016). No other significant sex differences in fidelity outcomes were observed. For the exercise intervention, reasons for non-attendance were documented on two occasions only (illness), and reasons for non-compliance were documented on six occasions (feeling unwell [e.g., dizziness, nausea], *n* = 4; knee pain, *n* = 1; punctuality, *n* = 1). Overall, 21%, 50%, and 29% of participants in the intervention reported RPEs below, within, and above the prescribed target range (moderate-intensity: RPE 12–13; high-intensity: RPE 14–17) [23], respectively. The average RPE for the moderate-intensity and high-intensity interventions were 12 (males: 11; females: 13) and 15.5 (males: 15; females: 16), respectively.

#### 3.2.3. Safety

Overall, 36 minor adverse events in ten participants (five males, five females) were documented throughout the study in 29% of the total sample. In the intervention group, nine participants (38%) experienced minor adverse events, with a total of 35 events recorded. One participant in the control group experienced one minor adverse event. These events occurred more frequently in females (0.08% versus 0.03%; *p* = 0.003). However, a considerable number of events (*n* = 17) occurred in one female participant in the aerobic training group. Adverse events included post-exercise hypoglycemia (blood glucose level ≤ 3.9 mmol/L; *n* = 26, 72%), hyperglycemia (blood glucose level ≥ 13.9 mmol/L; *n* = 4, 11%), feeling unwell (*n* = 4, 11%), knee pain (*n* = 1, 3%), and pre-exercise hypoglycemia (*n* = 1, 3%). Post-exercise hypoglycemia occurred in five participants and was most frequent in those taking insulin and/or two or more anti-hyperglycemic medications. Five adverse events resulted in non-commencement of exercise (hyperglycemia, *n* = 4; hypoglycemia, *n* = 1) and session termination (feeling unwell, *n* = 4; knee pain, *n* = 1), respectively.

### 3.3. Exploratory Sex-Specific Outcomes

#### 3.3.1. Arterial Health

Baseline values, within-group changes, and sex differences are presented in Table 3. A significant sex-by-treatment interaction was observed for changes in PWV and brachial SBP. Post-hoc analyses revealed that females in the intervention group experienced a larger reduction in brachial SBP than male counterparts (9 mmHg, 95%CI (3, 15); *p* = 0.011), and larger reductions in PWV and brachial SBP than female controls (4.2 m/s, 95%CI [2.1, 6.3] and 19 mmHg, 95%CI (5,34), respectively; both *p* < 0.001). No other significant sex-by-treatment interactions were observed. However, the sex difference in CSP change following aerobic training (10 mmHg, 95%CI (0, 21); *p* = 0.310) was clinically meaningful.

**Figure 2:**
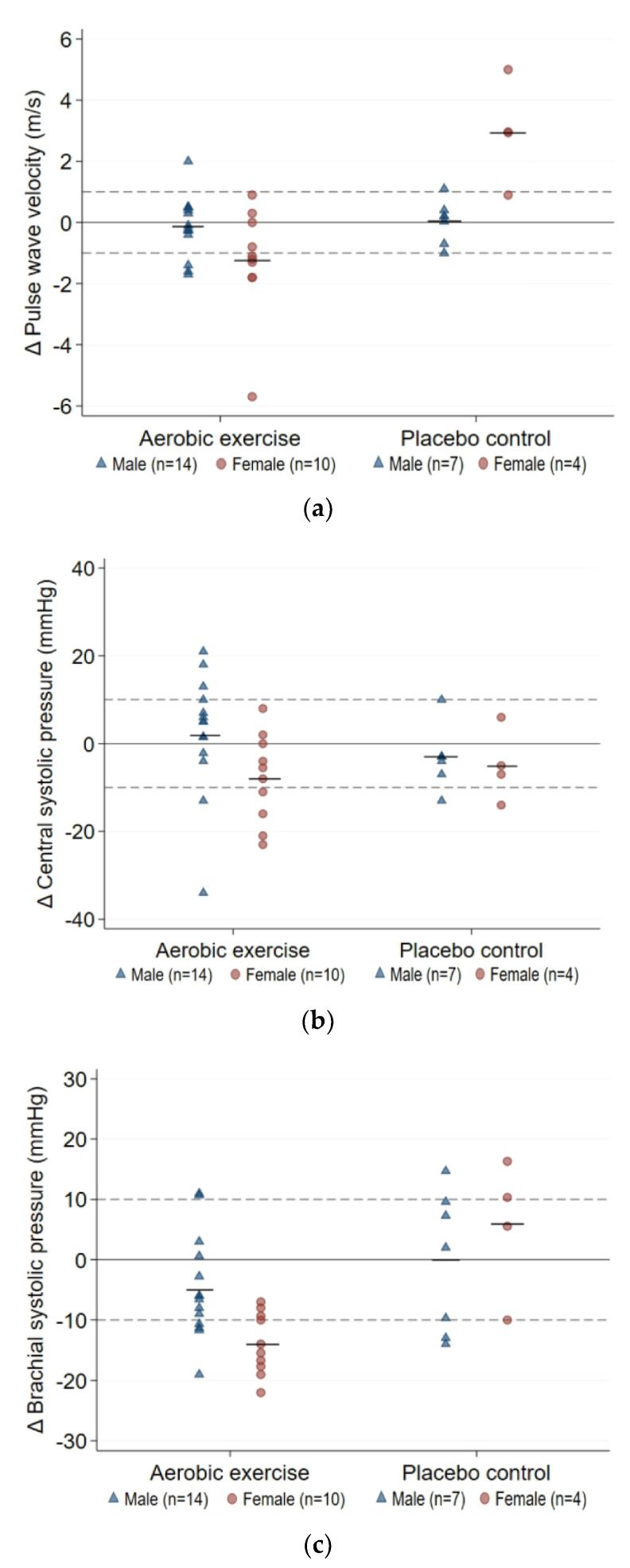
Individual 12-week changes in arterial health outcomes: (**a**) pulse wave velocity; (**b**) central systolic pressure; (**c**) brachial systolic pressure. Solid reference line: mean change from baseline; dashed reference line: minimal clinically important difference.

A higher proportion of females experienced clinically meaningful improvements in PWV (≥1 m/s [24]; 60% versus 21%, Figure 2a), central systolic pressure (≥10 mmHg [25]; 40% versus 14%, Figure 2b), and brachial SBP (≥10 mmHg [26]; 60% versus 29%, Figure 2c).

#### 3.3.2. Cardiometabolic Health

A significant sex-by-treatment interaction was observed for the change in waist circumference. Post-hoc analyses showed a greater reduction among females in the intervention group (*p* < 0.001, Table 4). No other statistically significant sex-by-treatment interactions were observed. Modest but non-significant improvements were observed for all other outcomes among females in the intervention group; improvements were clinically meaningful for HbA1c, FBG, and waist circumference (Table 4).

## 4. Discussion

Our secondary analyses of a randomized controlled trial examined the feasibility of assessing sex differences in the effects of aerobic exercise training on markers of arterial health and cardiometabolic risk. Female-specific recruitment and global adherence challenges indicate a need to include sex-specific strategies in future study designs, and hypothesis generating exploratory data describing sex-specific outcomes address a current lack of data for sample size calculations.

### 4.1. Feasibility Outcomes

While recruitment of adults with T2D into exercise trials can be challenging due to additional safety and medical concerns [27], our recruitment rate was lower than previous reports for exercise interventions lasting beyond 12 weeks in this population (17–78%) [28,29,30,31]. Direct comparisons between studies are difficult due to differences in eligibility criteria, recruitment strategies, and trial duration. However, these previous studies utilized at least four advertising strategies across multiple locations (e.g., diabetes support groups and clinics, community centers, pharmacies, hospitals and general practitioners, social media), whereas our study implemented two advertising strategies across three locations. Less reach within the community may have contributed to our lower recruitment rate.

Importantly, while reasons for non-participation were comparable between sexes, a greater proportion of females were ineligible or declined participation. This suggests future trials should invest in female-specific recruitment strategies to increase both trial efficiency and female representation in published data. A recent systematic review examining recruitment methods reported that recruitment via social media yielded greater interest from females than males [32]. While not specific to exercise trials, social media advertising may be an effective strategy for increasing recruitment of female participants. In The Geelong Osteoporosis Study, which recruited individuals aged 20 years and over, non-participation rates were higher among males than females (33% versus 23%, *p* < 0.001) [33]. Further, they found males more commonly reported time constraints and females more commonly reported frailty and reluctance of medical testing as reasons for non-participation. However, as with our study, disinterest was the most common reason for declined participation among both sexes [33], highlighting the importance of designing interventions that meet participant needs. People may be more likely to participate in research studies if they experience symptoms of the health condition [34,35]. Since many individuals with T2D are asymptomatic [36], recruitment strategies that increase awareness of the health condition and personal relevance of the study may promote recruitment and engagement in the trial.

Our findings reinforce previous research that showed time, travel limitations, and access to facilities are significant barriers to exercise participation in adults with T2D [37,38]. Strategies such as providing increased appointment flexibility (e.g., after hours, weekends) and multiple training locations or remote delivery, may improve recruitment rates. Finally, our retention rate was consistent with previous exercise trials lasting beyond 12 weeks in adults with T2D (86–97%) [28,31,39], indicating that a 10% inflation of sample size for attrition is necessary for future trials in this population [28,31,39].

We observed high attendance at exercise training sessions among both sexes, and while many exercise trials report similar rates (75–85%) [28,30,40], we believe this is the first study to examine sex differences in treatment fidelity among adults with T2D. Previous trials in cardiac rehabilitation commonly report higher attendance rates among males [41,42], which may be influenced by sex differences in barriers to participation. Grace et al. [43] showed females more frequently cite transportation, family responsibilities, and perceiving exercise as painful or tiring as barriers to exercise in cardiac rehabilitation, whereas males were more likely to cite work commitments. Our exclusion criteria may have contributed to the lack of sex difference in attendance rates by screening out some of these barriers (e.g., ability to commit to three sessions per week, proximity to training facility). Although females were significantly less compliant to the exercise intervention, and thus, had significantly poorer global adherence when compared to males, this was likely due to the influence of outliers in our small sample size. Nonetheless, these findings have important implications for the design of future trials investigating sex differences in this population, as well as the implementation of exercise interventions in real-world settings. In contrast to Grace et al. [43], we did not observe a sex difference in perceived exertion, which suggests that the intervention was generally well-tolerated by both sexes. However, it remains unclear whether higher volumes of high-intensity exercise would be equally as well tolerated. Given apparent sex differences in non-participation and adherence rates, future trials should consider sex-specific barriers to exercise when calculating required sample sizes to ensure potential sex differences in treatment effects are not underestimated.

Modest attendance rates in the control group suggests that participant acceptance of that treatment was low. A recent 6-month exercise trial in adults with T2D reported comparable attendance rates between the control and intervention groups (75% and 79%, respectively) [28]. While the primary aim of their study was to examine the efficacy of aerobic and resistance exercise on cognitive function, similar outcome measures to our trial were collected (central hemodynamics, anthropometry, blood samples, VO_2peak_), suggesting comparable participant burden between studies. One potential explanation for the higher attendance rate compared to our study is that their control treatment was designed to provide the same amount of participant involvement as the intervention (i.e., three sessions per week of stretching and mobility exercises). This may result in greater perceived benefit, motivation, and support. A sham-exercise control treatment involving similar contact time to the intervention may help to optimize control treatment fidelity. Minimizing perceived differences between treatments may also improve participant blinding.

A higher frequency of minor adverse events among females is likely explained by one outlying participant who accounted for 47% of all adverse events. Importantly, a lack of serious adverse events reinforces the safety of the intervention in this population [44,45]. A high proportion of post-exercise hypoglycemia was expected for this population and intervention [1], but highlights a need for future trials to include participant education regarding modification and timing of insulin dose and carbohydrate consumption with exercise. As fear of hypoglycemia can be a barrier to exercise [38], this strategy could increase adherence and foster self-efficacy to improve health outcomes.

### 4.2. Sex-specific Responses to Aerobic Training

To our knowledge, only one study has evaluated sex differences in arterial health following 12 weeks of aerobic training in adults with T2D [46]. Similar to Madden et al. [46], who reported no significant sex difference in PWV change (males: −15.1 ± 7.7%; females: −12.6 ± 6.6%), we did not observe a sex difference in PWV. However, females had a clinically meaningful reduction when compared to males (≥1 m/s), which has been associated with 14% and 15% lower risk of CVD events and mortality, respectively [24]. As we pooled data from two exercise interventions, direct comparisons between studies are limited. However, our exercise sessions were less frequent (three versus five times per week), and while our high-intensity protocol was of higher intensity (90%VO_2peak_ versus 60–75% heart rate reserve), it was considerably shorter in duration per session (4 min versus 40 min). This may suggest that males require a greater volume of aerobic exercise training to elicit improvements in central arterial stiffness. Baseline differences in CVD risk factors of males across both studies may further explain the greater improvement in the Madden et al. [46] male cohort. Our participants were younger (55 versus 71 years), with lower PWV (8.59 m/s versus 12.68 m/s) and SBP (131 mmHg versus 150 mmHg). A more favourable risk profile may reduce the capacity for improvement. However, Madden et al. [46] did not stratify baseline measures by sex, making it difficult to confirm this suggestion. This reinforces the need for deliberate and thoughtfully designed studies when researching sex differences.

In our study, the improvement in PWV among aerobically trained females was accompanied by modest-to-large reductions in central and peripheral pressures, suggesting that exercise may induce systemic vascular adaptations in females. The within-group effect on brachial systolic pressure and between-sex effect on central systolic pressure correspond with ~20% and 10% reductions in acute CVD events risk, respectively [25,26]. This may be explained by relatively good arterial health among female participants (PWV ≤ 10 m/s) [24]. Since healthy females exhibit greater local vasodilatory responses to exercise than males [12,13], reduced arterial resistance may have contributed to the superior effect on PWV among females. In contrast, males may have worse peripheral arterial and microvascular health, which may explain the increased AIx despite no increase in central PWV [47]. As cardiometabolic risk factors can increase arterial stiffness via pathological structural and functional vascular remodelling [48], the observed sex difference in arterial health outcomes may also be contributed to by the greater improvements for females in brachial systolic pressure and waist circumference, and clinically meaningful improvements in glucose control. These findings necessitate further investigation into the potential sex-specific benefits of exercise in this population to optimize outcomes in both sexes.

### 4.3. Strengths and Limitations

Use of gold standard data collection methods means our secondary analysis used precise and reliable data, and a placebo control treatment reduced performance bias and the likelihood that the observed sex-differences were due to chance. The small sample size is a major limitation, particularly the relatively low recruitment of female participants, which limits interpretation of between-sex treatment effects as exploratory and hypothesis generating. Nonetheless, we have identified several key feasibility considerations that can inform the design of larger trials to robustly compare effects between males and females. Pooling two exercise training groups precluded exploring sex-specific dose responses. However, a lack of differences in cardiometabolic health outcomes between these groups in the original randomized controlled trial suggests such effects were unlikely. Though imputation of incorrect or missing data may have increased the risk of type-1 error [49], the group mean method used was a conservative approach selected to mitigate this risk. Lastly, as with any exercise study, those who are more health conscious or motivated to exercise may be more likely to participate. Therefore, our findings may not be generalizable to all adults with T2D.

## 5. Conclusions

We found hypothesis generating signals that effects of aerobic exercise training on arterial and cardiometabolic health outcomes may differ between males and females with T2D, but recruitment and treatment fidelity are critical considerations for the design of robust experimental studies to test this. Strategies to increase female recruitment efficiency and treatment adherence will be required to optimize study feasibility and address the longstanding underrepresentation of females in research evidence.

## Figures and Tables

**Table 1:** Feasibility outcome definitions.

Outcome	Measurements
Recruitment	Number of enrolled-participants versus total-screened participants
	Reasons for non-participation
Retention	Number of participants available at follow-up
	Reasons for loss to follow-up
Treatment Fidelity	Attendance: proportion of prescribed sessions attended Compliance: proportion of aerobic training sessions attended with completion of full prescribed duration
	Global adherence: proportion of participants with ≥70% attendance and ≥70% compliance Perceived exertion: mean RPE across attended sessions
Safety	Number and severity of adverse events across attended sessions

**Table 2:** Baseline characteristics of participants.

Demographic	Aerobic Exercise (*n* = 24)		Placebo Control (*n* = 11)	
	Males (*n* = 14)	Females (*n* = 10)	Males (*n* = 7)	Females (*n* = 4)
Age (years)	57 ± 2	56 ± 2	51 ± 4	53 ± 2
T2D duration (years)	7 ± 2	11 ± 2	6 ± 2	9 ± 4
HbA1c (%)	6.8 ± 0.3	7.5 ± 0.6	7.8 ± 0.7	7.2 ± 0.7
BMI (m/kg^2^)	36.0 ± 1.5	35.8 ± 1.3	36.6 ± 1.9	34.4 ± 3.8
Weight (kg)	110.1 ± 4.3	94.3 ± 4.6	114.0 ± 6.0	92.1 ± 6.0
Waist circumference (cm)	118.4 ± 3.5	114.3 ± 3.4	122.5 ± 4.2	105.1 ± 5.6
VO_2peak_ (ml/kg/min)	23.8 ± 1.1	17.7 ± 0.7	21.4 ± 1.9	18.1 ± 5.0
SBP (mmHg)	132 ± 4	130 ± 4	135 ± 9	111 ± 6
DBP (mmHg)	81 ± 1	78 ± 2	86 ± 6	79 ± 4
HR (bpm)	66 ± 2	71 ± 3	77 ± 6	78 ± 4
Medications, *n (%)*				
Anti-hyperglycemic	13 (37)	10 (29)	7 (20)	3 (9)
Anti-hypertensive	10 (29)	4 (11)	5 (14)	1 (3)
Lipid lowering	10 (29)	5 (14)	6 (17)	1 (3)

T2D, type 2 diabetes; HbA1c, glycated hemoglobin; BMI, body mass index; VO_2peak_, cardiorespiratory fitness; SBP, systolic blood pressure; DBP, diastolic blood pressure; HR, heart rate.

**Table 3:** Mean baseline arterial health outcomes, within-group changes relative to baseline, and net sex differences per intervention.

	Aerobic Exercise			Placebo Control			Interaction Effect (Group × Sex)
	Males	Females	Net Sex Difference	Males	Females	Net Sex Difference	
PWV (m/s)							
Baseline	8.89 ± 0.43	8.28 ± 0.28		9.09 ± 0.60	6.55 ± 3.05		
Δ12-week	−0.12 (−0.69, 0.44)	−1.25 (−2.54, 0.04)	1.13 (−0.06, 2.31)	0.03 (−0.61, 0.68)	2.95 (0.29, 5.61)	−2.92 (−4.51, −1.33)	***p* < 0.0001**
AIx (%)							
Baseline	22.4 ± 1.9	32.6 ± 5.2		25.9 ± 4.2	31.3 ± 5.7		
Δ12-week	5.7 (−1.1, 12.5)	0.3 (−8.9, 9.5)	5.4 (−5.1, 15.9)	0.9 (−7.2, 8.9)	9.7 (1.4, 17.9)	−8.8 (−19.8, 2.1)	*p* = 0.098
AIx@75 (%)							
Baseline	18.0 ± 1.9	26.8 ± 4.0		27.0 ± 3.9	32.8 ± 7.0		
Δ12-week	8.6 (2.2, 15.0)	0.67 (−7.9, 9.2)	7.9 (−1.9, 17.8)	0.43 (−7.1, 8.0)	1.33 (−9.5, 12.2)	8.8 (−7.3, 24.9)	*p* = 0.272
AP (mmHg)							
Baseline	9 ± 1	15 ± 3		11 ± 2	15 ± 5		
Δ12-week	3 (−1, 12)	−1 (−5, 4)	4 (−2, 9)	1 (−4, 7)	0 (−11.30, 10.80)	0 (−9, 9)	*p* = 0.999
PP (mmHg)							
Baseline	39 ± 2	44 ± 3		41 ± 3	36 ± 6		
Δ12-week	1 (−3, 6)	−4 (−10, 2)	5 (−2, 12)	0 (−7, 7)	−4 (−18, 9)	4 (−7, 16)	*p* = 0.862
MAP (mmHg)							
Baseline	97 ± 3	98 ± 4		109 ± 7	95 ± 8		
Δ12-week	2 (−4, 9)	−3 (−10, 4)	5 (−4, 14)	−3 (−10, 3)	−2 (−8, 4)	−1 (−10, 8)	*p* = 0.253
CSP (mmHg)							
Baseline	121 ± 3	123 ± 5		132 ± 8	118 ± 12		
Δ12-week	2 (−5, 10)	−8 (−15, −1)	10 (0, 21)	−3 (−10, 3)	−5 (−19, 8)	2 (−9, 12)	*p* = 0.310
CDP (mmHg)							
Baseline	82 ± 2	81 ± 4		92 ± 6	82 ± 6		
Δ12-week	1 (−4, 7)	−2 (−9, 9)	3 (−5, 12)	−3 (−11, 5)	−1 (−3, 2)	−2 (−13, 8)	*p* = 0.256
SBP (mmHg)							
Baseline	132 ± 4	130 ± 4		135 ± 9	111 ± 6		
Δ12-week	−5 (−10, 0)	−14 (−18, −10)	9 (3, 15)	0 (−11, 10)	6 (−12, 23)	−6 (−22, 10)	***p* = 0.004**
DBP (mmHg)							
Baseline	81 ± 1	78 ± 2		86 ± 6	79 ± 4		
Δ12-week	−3 (−6, 1)	−1 (−7, 4)	−1 (−7, 4)	−4 (−14, 5)	−3 (−8, 3)	−2 (−14, 11)	*p* = 0.939
HRrest (bpm)							
Baseline	66 ± 2	71 ± 3		77 ± 6	78 ± 4		
Can removeΔ12-week	2 (−3, 6)	0 (−7, 7)	2 (−6, 9)	4 (−3, 11)	−9 (−20, 3)	13 (2, 23)	*p* = 0.068

Data are: baseline mean ± standard deviation; within-group mean change (95% confidence interval); net sex difference (95% confidence interval) was calculated by subtracting within-group change for females from that of males. PWV, pulse wave velocity; AIx, augmentation index; AIx@75HR, augmentation index normalized to heart rate; AP, augmentation pressure; MAP, mean arterial pressure; CSP, central systolic pressure; CDP, central diastolic pressure; SBP, systolic brachial pressure; DBP, diastolic brachial pressure; HRrest, heart rate at rest.

**Table 4:** Mean baseline cardiometabolic health indicators, within-group changes relative to baseline, and net sex differences per intervention.

	Aerobic Exercise			Placebo Control			Interaction Effect (Group × Sex)
	Males	Females	Net Sex Difference	Males	Females	Net Sex Difference	
VO_2peak_ (mL/kg/min)							
Baseline	23.82 ± 3.92	17.67 ± 2.34		21.35 ± 5.14	18.07 ± 4.96		
Δ12-week	0.76 (−1.00, 2.53)	2.72 (0.11, 5.34)	−1.96 (−4.81, 0.80)	−1.76 (−5.21, 1.70)	−0.63 (−1.49, 0.23)	−1.13 (−5.47, 3.22)	*p* = 0.734
BMI (kg/m^2^)							
Baseline	35.99 ± 1.45	35.78 ± 1.31		36.57 ± 1.86	34.40 ± 3.75		
Δ12-week	0.36 (−0.32, 1.04)	−0.17 (−0.76, 0.41)	0.53 (−0.36, 1.43)	0.61 (−0.61, 1.82)	0.05 (−0.49, 0.60)	0.55 (−0.99, 2.10)	*p* = 0.981
Weight (kg)							
Baseline	110.13 ± 4.27	94.33 ± 4.56		113.98 ± 5.97	92.13 ± 5.98		
Δ12-week	1.13 (−0.93, 3.18)	−0.45 (−1.88, 0.99)	1.58 (−1.02, 4.17)	1.92 (−1.88, 5.71)	0.07 (−1.36, 1.49)	1.85 (−2.96, 6.66)	*p* = 0.890
Waist circumference (cm)							
Baseline	118.4 ± 3.5	114.3 ± 3.4		122.5 ± 4.2	105.1 ± 5.6		
Δ12-week	−1.9 (−3.3, −0.5)	−5.8 (−7.8, −3.7)	3.8 (1.6, 6.1)	−1.5 (−3.8, 0.9)	1.3 (−3.4, 6.0)	−2.8 (−6.5, 1.0)	***p* = 0.002**
HbA1c (%)							
Baseline	6.83 ± 0.2	7.79 ± 1.89		7.52 ± 1.74	7.23 ± 0.74		
Δ12-week	0.04 (−0.35, 0.44)	−0.64 (−1.49, 0.22)	0.68 (−0.12, 1.48)	0.30 (−0.41, 1.01)	0.20 (−0.27, 0.67)	0.10 (−0.82, 1.02)	*p* = 0.373
FBG (mmol/L)							
Baseline	7.36 ± 0.49	8.20 ± 0.97		10.26 ± 1.83	6.95 ± 0.45		
Δ12-week	0.44 (−0.45, 1.34)	−1.15 (−2.42, 0.12)	1.59 (0.18, 3.01)	−0.24 (−4.93, 4.45)	0.17 (−0.84, 1.18)	−0.41 (−6.30, 5.48)	*p* = 0.355
Fasting insulin (mU/L)							
Baseline	11.07 ± 1.88	25.60 ± 10.66		14.00 ± 1.20	13.25 ± 5.36		
Δ12-week	2.56 (0.25, 4.88)	−0.45 (−5.52, 4.62)	3.01 (−1.70, 7.72)	−1.33 (−5.46, 2.79)	3.33 (−3.08, 9.74)	−4.67 (−10.79, 1.46)	*p* = 0.057
Total cholesterol (mmol/L)							
Baseline	4.17 ± 0.17	4.76 ± 0.24		4.23 ± 0.32	4.95 ± 0.49		
Δ12-week	0.15 (−0.32, 0.61)	−0.35 (−1.08, 0.39)	0.49 (−0.29, 1.27)	0.06 (−0.45, 0.56)	0.03 (−0.24, 0.30)	0.02 (−0.62, 0.67)	*p* = 0.444
LDL (mmol/L)							
Baseline	2.36 ± 0.20	2.77 ± 0.20		2.03 ± 0.24	2.88 ± 0.40		
Δ12-week	0.04 (−0.36, 0.43)	−0.32 (−0.94, 0.30)	0.35 (−0.30, 1.01)	−0.08 (−0.47, 0.30)	−0.13 (−0.53, 0.26)	0.05 (−0.48, 0.58)	*p* = 0.434
HDL (mmol/L)							
Baseline	1.02 ± 0.05	1.28 ± 0.08		1.06 ± 0.10	1.43 ± 0.13		
Δ12-week	−0.01 (−0.08, 0.06)	0.07 (−0.07, 0.20)	−0.07 (−0.20, 0.06)	−0.01 (−0.08, 0.05)	−0.07 (−0.27, 0.13)	0.05 (−0.08, 0.18)	*p* = 0.229
Triglycerides (mmol/L)							
Baseline	1.69 ± 0.19	1.54 ± 0.19		1.87 ± 0.28	1.40 ± 0.15		
Δ12-week	0.32 (0.03, 0.61)	−0.21 (−0.59, 0.18)	0.53 (0.08, 0.97)	0.38 (−0.38, 1.15)	0.47 (−0.47, 1.41)	−0.08 (−1.16, 0.99)	*p* = 0.185
hsCRP (mg/L)							
Baseline	2.47 ± 0.50	8.03 ± 2.53		2.81 ± 0.68	6.85 ± 2.71		
Δ12-week	0.21 (−0.46, 0.89)	−3.05 (−6.33, 0.24)	3.26 (0.62, 5.89)	2.34 (−2.19, 6.87)	−1.53 (−5.33, 2.26)	3.88 (−2.12, 9.88)	*p* = 0.820

Data are: baseline mean ± standard deviation; within-group mean change (95% confidence interval); net sex difference (95% confidence interval) was calculated by subtracting within-group change for females from that of males for each intervention. VO_2peak_, peak oxygen consumption; BMI, body mass index; HbA1c, glycated hemoglobin; FBG, fasting blood glucose; LDL, low-density lipoprotein cholesterol; HDL, high-density lipoprotein cholesterol; hsCRP, high-sensitivity C-reactive protein.

**Table 5:** Number and proportion (%) of participants randomized compared to total screened.

	Male, *n* (%)	Female, *n* (%)	Total, *n* (%)
Screened	120 (43)	161 (57)	281 (100)
Unable to be contacted	21 (7)	31 (11)	50 (18)
Declined participation	35 (13)	50 (18)	87 (31)
Ineligible	43 (15)	66 (24)	109 (39)
Total randomized	21 (7)	14 (5)	35 (12)

## Data Availability

The data presented in this study are available on request from the corresponding author. The data are not publicly available due to privacy.
